# Natural Organic Materials Based Memristors and Transistors for Artificial Synaptic Devices in Sustainable Neuromorphic Computing Systems

**DOI:** 10.3390/mi14020235

**Published:** 2023-01-17

**Authors:** Md Mehedi Hasan Tanim, Zoe Templin, Feng Zhao

**Affiliations:** Micro/Nanoelectronics and Energy Laboratory, School of Engineering and Computer Science, Washington State University, Vancouver, WA 98686, USA

**Keywords:** natural organic materials, memristor, transistor, artificial synaptic device, neuromorphic computing, synaptic functions

## Abstract

Natural organic materials such as protein and carbohydrates are abundant in nature, renewable, and biodegradable, desirable for the construction of artificial synaptic devices for emerging neuromorphic computing systems with energy efficient operation and environmentally friendly disposal. These artificial synaptic devices are based on memristors or transistors with the memristive layer or gate dielectric formed by natural organic materials. The fundamental requirement for these synaptic devices is the ability to mimic the memory and learning behaviors of biological synapses. This paper reviews the synaptic functions emulated by a variety of artificial synaptic devices based on natural organic materials and provides a useful guidance for testing and investigating more of such devices.

## 1. Introduction

Currently, the von Neumann bottleneck limits conventional computing from meeting the demands of energy-intensive applications such as blockchain, artificial intelligence, etc., i.e., the energy issue. Moreover, computing hardware components, when disposed, produce electronic waste that can be toxic and not biodegradable and, therefore, harmful to the environment, i.e., the environment issue. A potential solution to address the energy issues is by emerging neuromorphic computing which mimics the brain by processing and storing data in the same unit [1] for energy-efficient operation. Among different types of electronic devices, memristor and transistor based artificial synaptic devices have emerged as potential hardware components for neuromorphic computing systems because of their capabilities of mimicking synaptic connections between neurons. These devices, if made from environmentally friendly natural organic materials [2], have also the potential to solve the environment issue. 

**Figure 1:**
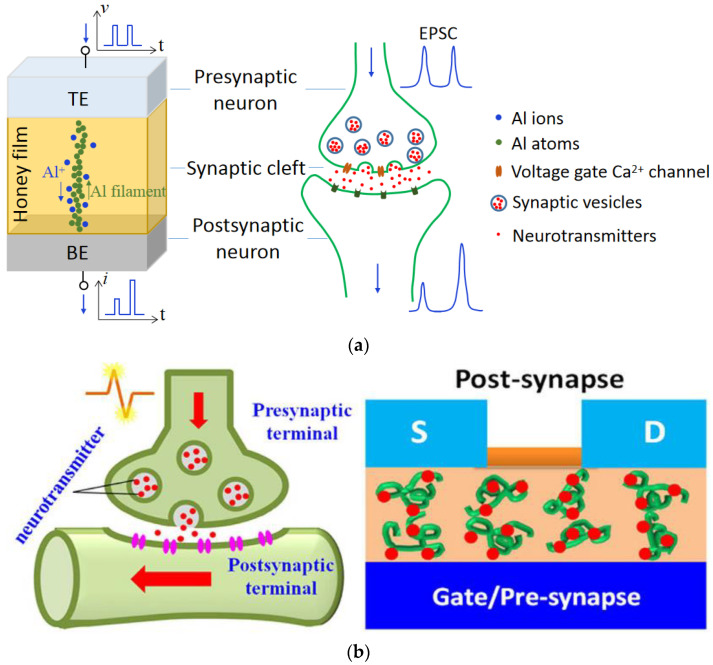
Resemblance to biological synapses of artificial synaptic devices based on (**a**) memristor [20] and (**b**) transistor [43].

A variety of natural organic materials, mainly protein and carbohydrates such as chitosan [3,4,5,6], Aloe Vera and polysaccharide [7,8,9,10,11,12,13], zein from maize [14], gelatin [15], honey [16,17,18,19,20,21,22], lignin [23,24], collagen [25], trypsin [26], ι-carrageenan [27,28], dextran [29,30] sodium alginate [31], wood-derived cellulose nanopapers [32], biomass-based hydrogel [33], casein [34], pectin [35,36], carotene [37], lipid [38], natural acidic polyelectrolyte [39], chicken albumen [40,41], etc., are currently under investigation for making memristor- and transistor-based artificial synaptic devices. These natural organic materials have the advantages of being renewable, sustainable, biodegradable, environmentally friendly, etc., making them an attractive alternative to other inorganic metal oxide and polymer materials [42] for artificial synaptic devices. Neurons and synapses are basic building blocks of the human brain, with memory and learning behaviors achieved by modulating ion flows in them, which results in the transmission of neurotransmitters from the presynaptic neuron to the postsynaptic neuron. As shown in Figure 1, both synaptic memristor [20] and transistor [43] resemble biological synapses. Memristor has the metal-insulator-metal (MIM) structure that resembles the biological synapse as in Figure 1a, where the top electrode is analogous to the presynaptic neuron, the bottom electrode to the post-synaptic neuron, and the memristor thin film in between, which is processed from natural organic material, mimics the synaptic cleft. The current flow in the memristor emulates the excitatory postsynaptic current (EPSC), which is used to represent synaptic strength and elicited by stimulation in the presynaptic neuron and recorded in the postsynaptic neuron. In a synaptic transistor, the bottom-gate electrode and top source/drain electrodes with the channel layer are analogous to the presynaptic neuron and the postsynaptic neuron, respectively, as shown in Figure 1b. The natural organic film is regarded as the synaptic cleft and the channel current as EPSC.

Artificial synaptic devices need to be capable of emulating biological synaptic plasticity [44], one of the most important neurochemical foundations of learning and memory. Synaptic plasticity is the ability to strengthen or weaken synapses over time in response to increase or decrease in their activity. Important forms of synaptic plasticity include short-term plasticity (STP), long-term plasticity (LTP), neural facilitation and depression, spike-timing-dependent plasticity (STDP), spike-rate-dependent plasticity (SRDP), etc. In STP, the excitatory postsynaptic potentials (EPSPs) in synapses are directly affected by presynaptic spikes [45,46]. The basic STP includes short-term depression and short-term potentiation, and neural facilitation and depression [45,46]. STP can transit to LTP due to the structural change in synapse [47] such as the size increase of the synapse, additional release of neurotransmitters by the presynaptic neuron, and higher gain of receptors by the postsynaptic neuron [48]. STDP and SRDP play important roles in the development and refinement of neuronal circuits during brain development [49,50,51]. They are responsible for learning processes in the brain and retaining new information in neurons, and serve as the synaptic weight modification rule for neural network [52] via learning protocols such as the Hebbian learning rule that “those who fire together, wire together” [53].

This paper reviews different forms of synaptic behaviors that have been emulated by memristor- and transistor-based artificial synaptic devices made from natural organic materials.

## 2. Analog Memristive Behaviors

Analog memristive behaviors are the fundamental property of artificial synaptic devices for emulating synaptic plasticity. To characterize analog memristive properties, the device is tested under DC voltage sweeps, with voltage controlled carefully to prevent direct digital switching. During the test, consecutive voltage sweeps are first applied to make the current in the device increase in an analog fashion and then consecutive voltage sweeps in the opposite polarity are applied so that the current level decreases but in the same analog fashion. Such analog fashion of increase and decrease in current is similar to synaptic potentiation and depression of biological synapses. Memristors based on a variety of natural organic materials have demonstrated analog memristive behaviors.

**Figure 2:**
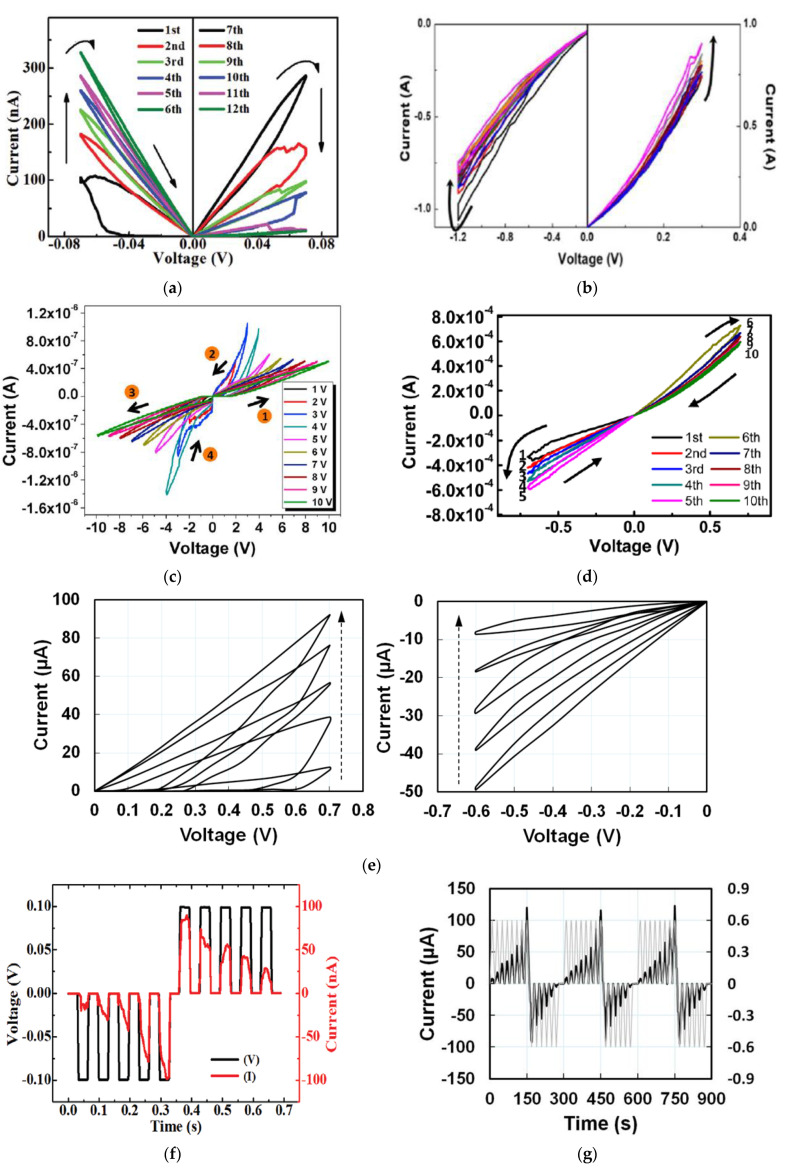

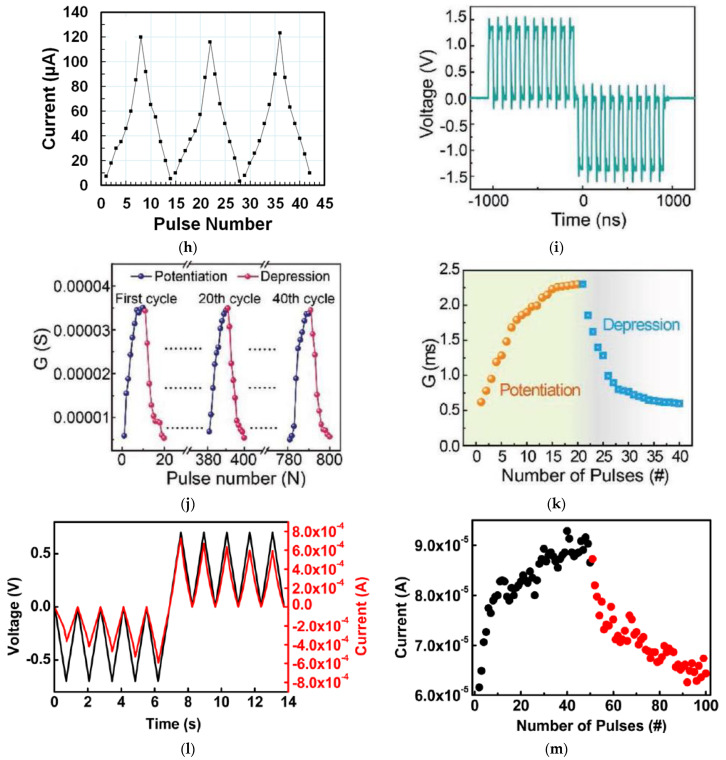
Analog switching characteristics of synaptic memristors based on natural organic materials of (**a**) cellulose nanocrystal [54], (**b**) zein from maize [14], (**c**) trypsin [26], (**d**) lignin [24], and (**e**) honey [17]. Current-time responses with applied voltage pulses of opposite polarity for memristors based on (**f**) cellulose nanocrystal [54], (**g**,**h**) honey [17], (**i**–**k**) bombyx mori silk [55], and (**l**,**m**) lignin [24]. In (**k**,**m**), # is the number of pulses. In (**m**), black dots are for potentiation and red dots for depression.

When six consecutive positive and negative voltage sweeps of ±0.07 V were applied on a cellulose-memristor [54], as shown in Figure 2a, the absolute current levels gradually increased from curve 1 to 6 after negative voltage sweeps, and then gradually decreased after each positive sweep from curve 7–12. Both increasing and decreasing curves followed the analog stepwise fashion. Potentiation and depression characteristics for a total of 10 consecutive sweep cycles in the DC sweep mode of a memristor based on zein [14] that is extracted from natural maize are shown in Figure 2b, in which the current gradually increased and decreased over each DC sweep. To avoid any abrupt change in the current, the voltages were chosen to be smaller than the threshold voltage. Besides cellulose and zein, memristors based on other natural organic materials such as honey [17], lignin [24], trypsin [26], collagen [25], dextran [29,30], chicken egg albumen [40], etc., have also demonstrated analog memristive characteristics. Some representative results are shown in Figure 2c–e.

Besides applying DC voltage sweeps to realize the continuous and gradual change in the current level, analog memristor behaviors were also investigated by transient electrical characterization by applying input voltage pulses to the device and recording the current response with time. Such pulse tests have been reported by memristors based on aloe polysaccharide [10], honey [17], lignin [24], dextran [30], chicken egg albumen [40], cellulose [54], bombyx mori silk [55], etc., as shown in Figure 2f–m. When 10 consecutive pulses (±0.10 V, 30 ms) with opposite polarities were applied on a cellulose-memristor [54], the increase of current values upon five successive negative bias pulses demonstrated the potentiation of the conductance states, while the decrease of current values upon five successive positive bias pulses showed the depression of the conductance of the device. A honey-memristor [17] was also tested by eight consecutive voltage pulses with a frequency of 0.05 Hz and amplitude of 0.6 V, first in positive polarity and followed by negative polarity. The time evolution and amplitude of the current under each voltage pulse are shown in Figure 2g,h. The amplitude of the current steadily increased with the positive voltage pulses, indicating synaptic potentiation. When the voltage pulses changed to negative polarity, amplitude of the current decayed until the last negative voltage pulse, a behavior analogous to synaptic depression. Three test cycles in Figure 2g,h proved that the honey-memristor has not only analog memristive behaviors to mimic biological synapses but also excellent repeatability.

## 3. Synaptic Plasticity

### 3.1. Short Term Memory and Long Term Memory

In neuroscience, short-term memory (STM) and long-term memory (LTM) are two forms of memory behaviors according to the retention time. The memory level in the human brain usually depends on the learning intensity and memory frequency. STM can be converted to LTM through a rehearsal learning process. STM and LTM have been widely studied in artificial synaptic devices based on natural organic materials [4,9,14,15,17,27,29,30,31,33,35,36,37,38,39,41,43,56,57], etc. They were tested by applying consecutive pulses (voltage or light) on the gate electrode of a transistor or one of the electrodes of a memristor as the external stimuli, and the nonvolatile current flow in the device was recorded as the memory level.

**Figure 3:**
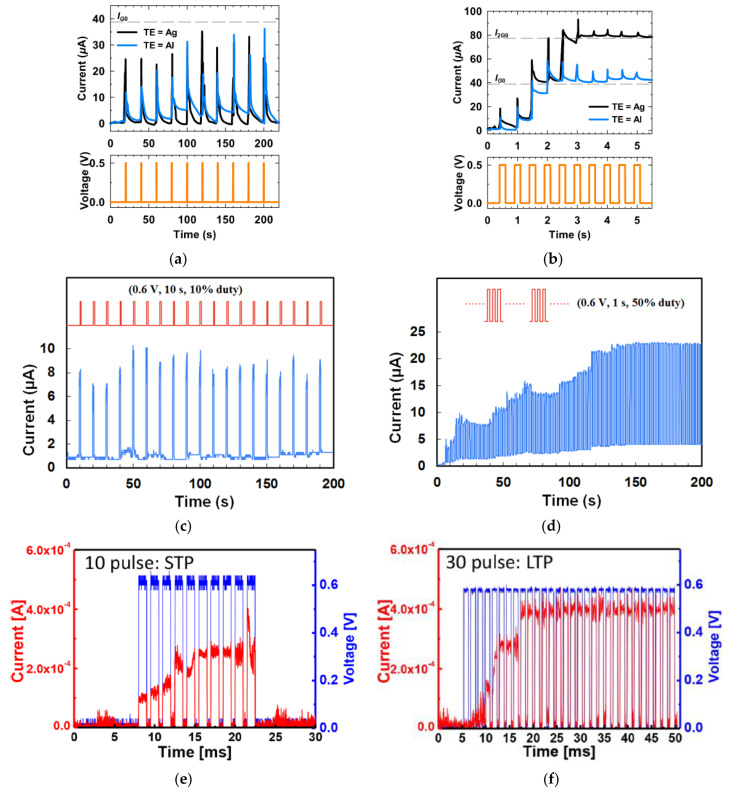
STM, LTM, and STM-to-LTM transition in synaptic devices based on (**a**,**b**) Aloe polysaccharide [9] and (**c**,**d**) honey [17] achieved by shortening the time interval between stimulation pulses and (**e**,**f**) ι-carrageenan [27] achieved by increasing the pulse number.

Figure 3a shows the current conduction behaviors of two Aloe polysaccharide memristors [9] with Ag and Al top electrode, respectively. When 10 pulses of 0.5 V with a uniform pulse width of 200 ms and pulse interval of 20 s were applied on both devices, the current flowing decayed almost instantaneously after each pulse, and the final current level did not increase after 10 pulses, leading to the STM, which was attributed to the weak filament formation by the stimuli. When the input pulse interval was shortened to 0.5 s, the current level in both devices increased clearly after each pulse, and after three or four pulses, the current stayed at a higher level so the increment in current was retained [Figure 3b], which is the LTM characteristic. Shortening pulse interval is an effective method to achieve STM-to-LTM transition, and it is also demonstrated by the honey-memristor [17], as shown in Figure 3c,d. Another effective method is increasing the number of pulses. When electrical pulses at a frequency of 667 Hz and an amplitude of 0.6 V were used as stimuli and applied on the ι-carrageenan-memristor [27], the device demonstrated STM after 10 of such pulses and transited to LTM after 30 pulses, as reported in Figure 3c,d.

**Figure 4:**
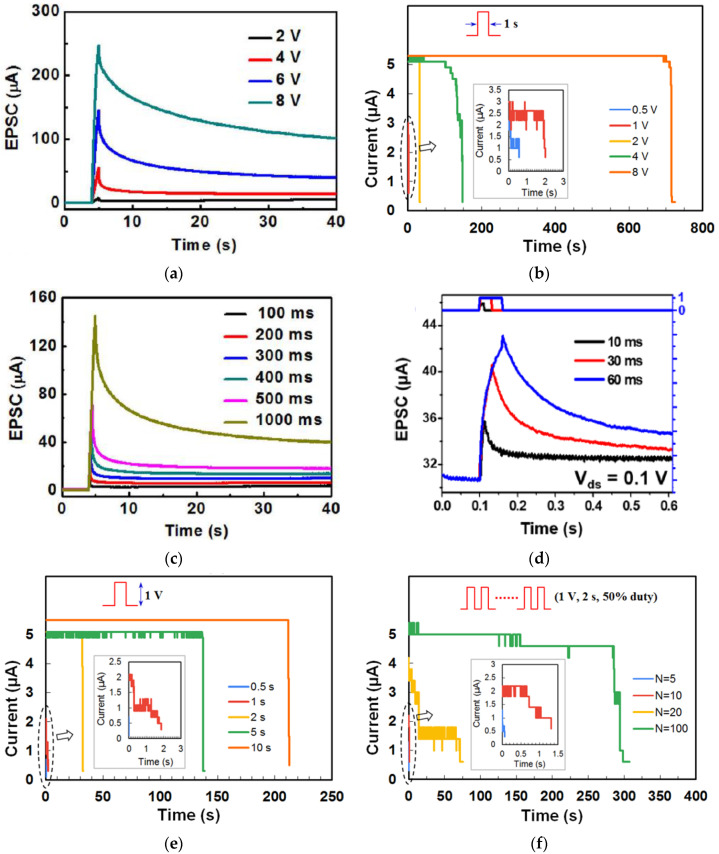

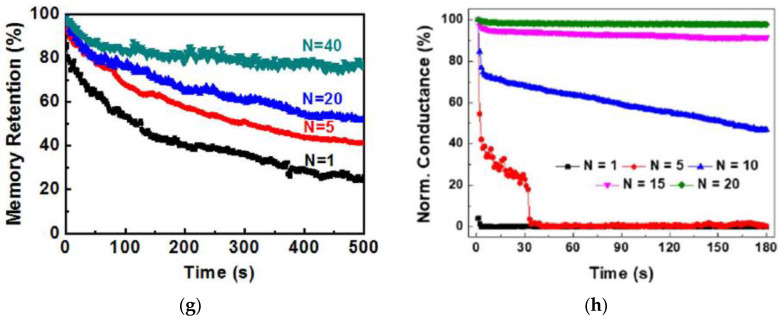
Current (memory) level and retention time affected by input pulse amplitude, which was demonstrated in (**a**) a chicken albumen transistor [43] and (**b**) a honey-memristor [17], pulse width, which was demonstrated by (**c**) a chicken albumen transistor [43], (**d**) a maltose-ascorbic acid electrolyte (MAE) gated transistor [56], and (**e**) a honey-memristor [17], and pulse number, which was demonstrated by (**f**) a honey-memristor [17], (**g**) a chicken albumen transistor [43], and (**h**) a zein-transistor [14].

The retention of current (memory level) in the device after stimulation is an important property for STM and LTM and was investigated by different stimulation parameters. Figure 4a shows the retention time of currents when input voltage pulses with the same 1s width but different amplitudes were applied on the gate of a chicken albumen-transistor [43]. The nonvolatile current increased clearly with the gate voltage pulse amplitude from 2 V to 8 V, indicating that a stronger gate pulse can lead to a higher memory level. The effect of pulse voltage amplitude on current retention was also investigated in a honey-memristor [17]. With the same 1s pulse width but increased voltage amplitude from 1 V to 8 V, the peak retention currents nearly doubled, and the retention time increased significantly from 2 s to 700 s, as shown in Figure 4b. Besides stimulation strength, pulse width can also increase current retention time. When the chicken albumen-transistor is tested by applying gate pulses with the same amplitude of 6.0 V but different pulse widths from 100 ms to 1 s [43], both peak and retention current increased clearly as in Figure 4c, indicating that the longer pulse width would also result in a higher memory level, which is similar to that observed in our brain [58]. A similar effect of pulse width was also demonstrated in a maltose-ascorbic acid electrolyte (MAE) gated transistor (Figure 4d) [56] and a honey-memristor (Figure 4e) [17]. Notably, not only are the input pulse amplitude and width, the current retention and memory level also increase with the pulse number, i.e., repeated stimulation. This effect was tested by applying a pulse train with the same amplitude and pulse width but different pulse numbers, with the retention current recorded after the last stimuli of each pulse train. As shown in Figure 4f–h, from the measurements on a honey-memristor [17], a zein-memristor [14], and a chicken albumen-transistor [43], the current level and retention time were greatly improved by increasing the number of stimulation pulses. This result is similar to that in a biological neural system where repeated rehearsal transforms short-term memory to long-term memory.

**Figure 5:**
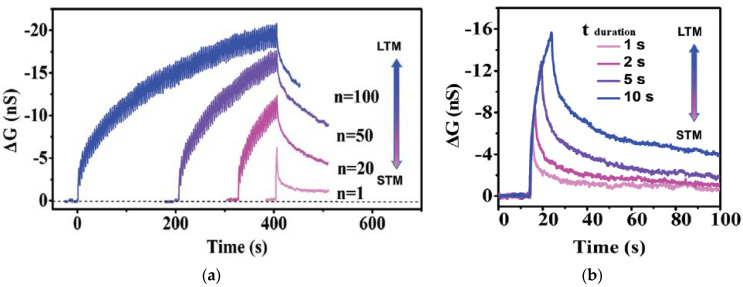
The STM-to-LTM transition induced by light pulses with increased (**a**) pulse number and (**b**) pulse width in a chlorophyll-a based synaptic transistor [57].

Besides electrical stimulation, optical pulses have also been tested on synaptic devices to achieve STM, LTM, and the transition from STM to LTM. Figure 5a,b shows the effect of optical pulse numbers and pulse widths on a chlorophyll-a based synaptic transistor [57]. Similar to electrical stimulation, optical pulse number and pulse width mimic the number of rehearsal occurrences and learning time, respectively. As shown in Figure 5a, the current levels increased with the number of optical pulses, indicating that the memory level in the device was strengthened by repetitive optical stimuli and the device achieved the STM-to-LTM transition. The optical pulse width also impacts the memory level. As depicted in Figure 5b, the recorded current peaks and retention times increased by increasing the optical pulse width.

**Figure 6:**
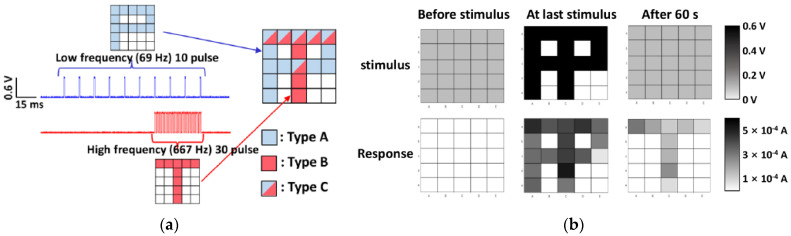
STM and LTM demonstrated by pattern memorization of a l-car artificial synapse 5 × 5 array [27] when two sets of stimuli with different pulse number and frequency were applied. (**a**) Letters of ‘P’ and ‘T’ are memorized on a 5 × 5 l-car memristor array by inputting the letter of ‘P’ for 10 times at a low frequency and ‘T’ for 30 times at a high frequency, respectively. (**b**) Applied stimulus and current response of the l-car memristor array before stimulus, at the last stimulus, and after 60 s.

STM and LTM have also been demonstrated via pattern memorization by a variety of natural organic synaptic device arrays based on chitosan [4], zein [14], ι-carrageenan [27], wool keratin [59], and chlorophyll-a/cellulose [60], etc., which proved the potential for the construction of neural networks. The representative results are shown in Figure 6 from a 5 × 5 ι-carrageenan artificial synapse array [27]. During the tests, two sets of stimuli were applied on the pixels to store two images, respectively, in which the first set consisted of 10 voltage pulses at a low frequency of 69 Hz and an amplitude of 0.6 V to form the “P” image, while the second set consisted of 30 voltage pulses at a high frequency of 667 Hz and an amplitude of 0.6 V for the “T” image. All 25 ι-carrageenan synapses were in the low conductance state (off-state) before the stimuli were applied. When the conductance of those ι-carrageenan synapses increased by the first stimuli set, the “P” image was stored temporarily in the synaptic array but forgotten after 60 s and returned back to the off-state, indicating STM. When the second stimuli sets with the higher strength was applied, ι-carrageenan synaptic pixels changed to the high conductance state (on-state) and retained after 60s with an elongated time, indicating LTM. This result proved that the transition from STM to LTM depends on input parameters such as the number and frequency of stimuli in ι- carrageenan synapses.

### 3.2. Paired Pulse Facilitation

Neural facilitation, also known as paired-pulse facilitation (PPF), is an important short-term plasticity [61] for neural tasks such as learning, information processing, auditory or visual source localization [3], etc. When a stimulus is applied on a presynaptic neuron, it causes an influx of produced ions, and as a result, neurotransmitters get released in the biological synapse when an action potential is achieved [45]. This process amplifies the synaptic transmission to the postsynaptic neuron for a certain time. The excited ion concentration requires time for the synapse to return to the original state prior to the first stimulation. If a second identical stimulus is applied before the synapse returns to its original state, the postsynaptic neuron response to the second stimulation is larger than its initial response. This effect is defined as PPF [23,24]. To emulate PPF, two stimulation pulses with a time interval mimicking presynaptic spikes are applied on the synaptic device, with the resulted current spikes (A_1_ and A_2_) in the device mimicking EPSC. The ratio of the absolute amplitudes of the first and second current spike, i.e., the PPF index (A_2_/A_1_), is used to quantify the PPF effect. Memristors and transistors based on a few natural organic materials such as gelatin [15], honey [19,20], lignin [24], ι-carrageenan [27], sodium alginate [31], biomass-based hydrogel [33], pectin [35,36], carotene [37], natural acidic polyelectrolyte [39], chicken albumen [43], silk [55], chlorophyl-a [57], wool keratin [59], cellulose [60], etc., have been reported to successfully mimic the PPF effect.

**Figure 7:**
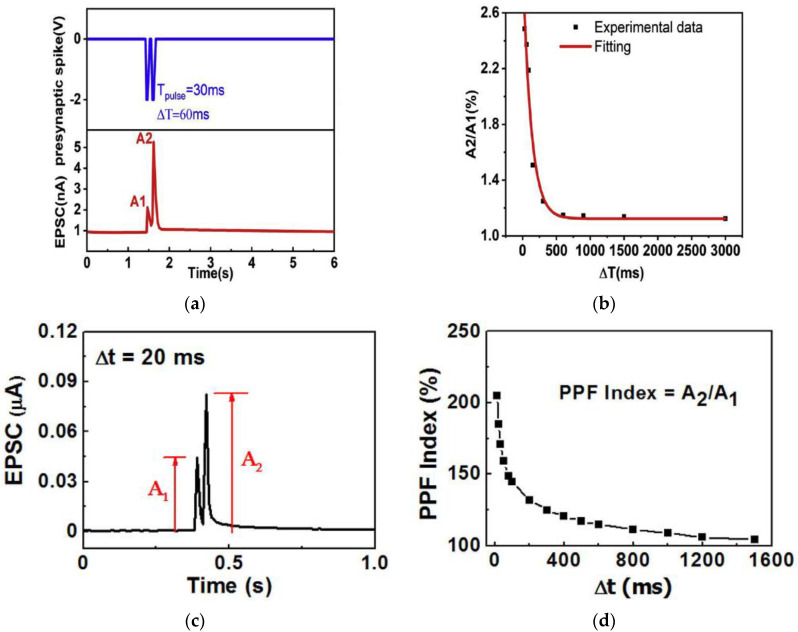

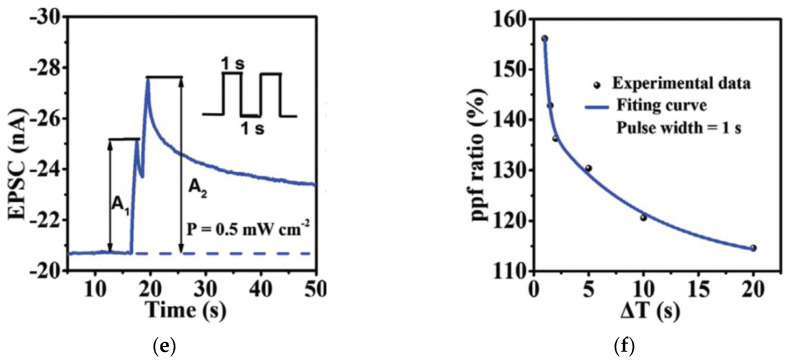
PPF characteristics and PPF index of synaptic transistors based on natural organic materials: (**a**,**b**) gelatin [15], (**c**,**d**) chicken albumen [43], (**e**,**f**) chlorophyll-a [57].

With movable ions in the gelatin-hydrogel electrolyte layer [15], gelatin-based synaptic transistor demonstrated PPF behaviors successfully. During the test, stimulation of two consecutive presynaptic voltage spikes (amplitude: −2 V, pulse width: 30 ms) and time interval (ΔT) were applied as a gate bias when drain-source voltage V_DS_ was fixed at −1 V. The ions triggered by the first spike did not have adequate time to diffuse back to the gelatin-hydrogel layer before the second spike arrived, to which the second EPSC was increased by the residual ions. As a result, the second EPSC (A_2_) was higher than the first EPSC (A_1_) as shown in Figure 7a, which is the PPF behavior observed in the biological system. With an increased ΔT, a decrease in the current was observed. A maximum PPF index (A_2_/A_1_) of ~250% was achieved at ΔT = 60 ms as in Figure 7b, and it gradually declined towards 100% for ΔT above 3000 ms. Emulation of PPF by a chicken albumen-based synaptic transistor [43] was tested by applying two successive presynaptic voltage spikes (amplitude: 0.5 V, pulse width: 10 ms) with the time intervals (Δt_Pre_) from 10 ms to 1500 ms. As shown in Figure 7c,d, the second EPSC is larger than the first EPSC as expected by PPF. The PPF index (A_2_/A_1_) reached the maximum value of ~205% at Δt_Pre_ = 10 ms and decreased gradually with the increase of Δt_Pre_, a similar behavior to the gelatin-transistor.

Besides electrical synaptic transistors stimulated by voltage spikes, photonic synaptic transistors based on natural organic materials have also reported the emulation of PPF behaviors by applying presynaptic optical pulses. In a chlorophyll-a based photonic synaptic transistor [57], when two consecutive excitatory optical pulses (wavelength: 665 nm, power: 0.5 mW/cm^2^, pulse width: 1 s) with an interval of 1 s were applied, the EPSC value (A_2_) stimulated by the second spike was larger than that of the first one (A_1_), as shown in Figure 7e, which confirmed the PPF behavior of the device. The PPF index (A2/A1) also decreased when the interval time increased [Figure 7f], similar to the electrical synaptic transistors.

**Figure 8:**
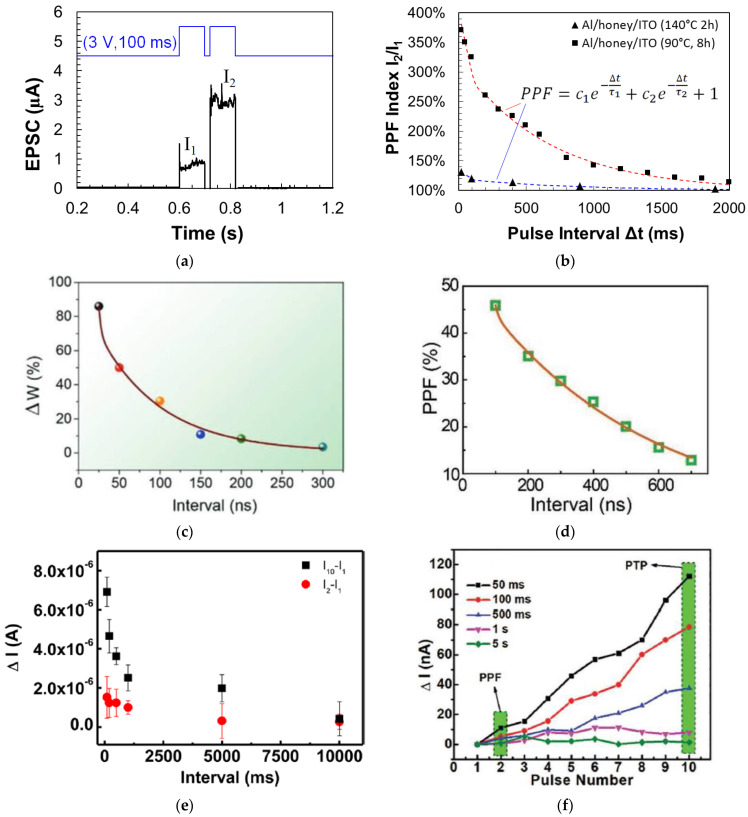
PPF characteristics and PPF index of synaptic memristors based on natural organic materials: (**a**,**b**) honey [20], (**c**) wool keratin [59], (**d**) silk [55], (**e**) lignin [24], and (**f**) cellulose [54]. In (**e**,**f**), PTP was also demonstrated.

Synaptic memristors based on a variety of natural organic materials have also demonstrated PPF behaviors. Figure 8a shows that when a pair of input voltage pulses (amplitude: 3 V, pulse width: 100 ms) with the time interval Δt of 20 ms were applied on a honey-memristor [20] with the Al top electrode, the amplitude of the second EPSC pulse was enhanced compared to the first EPSC pulse. The PPF index under the pulses with different time intervals were also measured and modeled by a double exponential decay: $=c_{1}e^{-\frac{\Delta t}{\tau_{1}}}+c_{2}e^{-\frac{\Delta t}{\tau_{2}}}+1$, where *c*_1_ and *c*_2_ are initial facilitation amplitude of the rapid phase and slow phase, and *τ*_1_ and *τ*_2_ are characteristic relaxation times of the rapid phase and slow phase, respectively. The PPF index and fitting curves on two honey-memristors with the honey films dried under two different conditions are shown in Figure 8b [20], with calculated values of *c*_1_, *c*_2_, *τ*_1_, and *τ*_2_ reported. Figure 8c,d show the PPF index by memristors based on silk [55] and wool keratin [59].

Similar to PPF, a post-tetanic potentiation (PTP) effect was reported by an aloe polysaccharide-memristor [10] and a lignin-memristor [24]. PTP represents the gradual increase in synaptic transmission when, instead of two stimulation spikes, multiple sequential stimulation spikes are applied within a short time. In this test, 10 consecutive stimulation voltage pulses were applied on the lignin-memristor, with EPSC recorded after each pulse. The changes in current were evaluated by the difference of the EPSC amplitude, where (*I*_2_–*I*_1_) is for PPF and (*I*_10_–*I*_1_) is for PTP. As represented in Figure 8e, both PPF and PTP results confirmed that synaptic weight in the lignin-memristor can be adjusted by controlling the spike rate when sequential spikes with the appropriate spike rate are applied on the device. The PTP effect was also demonstrated by a cellulose-memristor [54], as shown in Figure 8f, in which the increased current in the form of change in current (Δ*I*) was calculated by subtracting the current (*I_N_*, *N* = 1, 2, 3, 4, …, 10) from *I*_1_ with various intervals between input voltage spikes.

### 3.3. Spike-Timing-Dependent Plasticity

**Figure 9:**
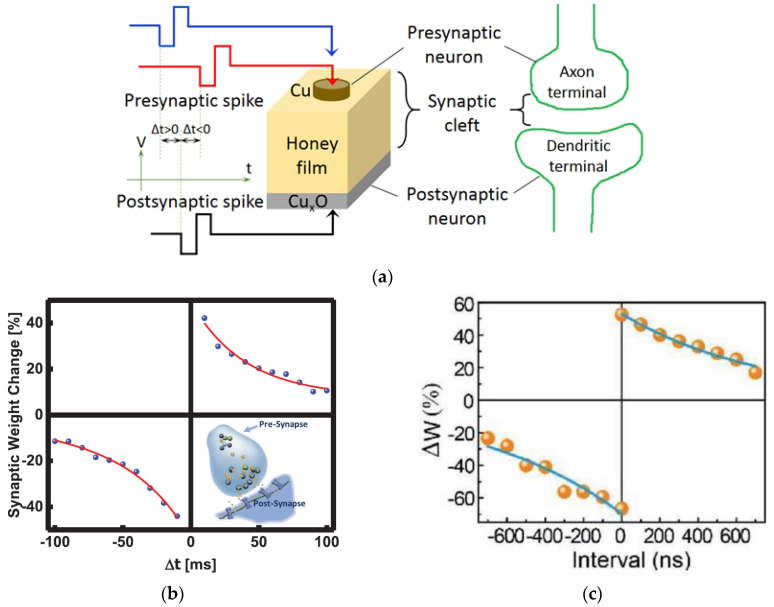

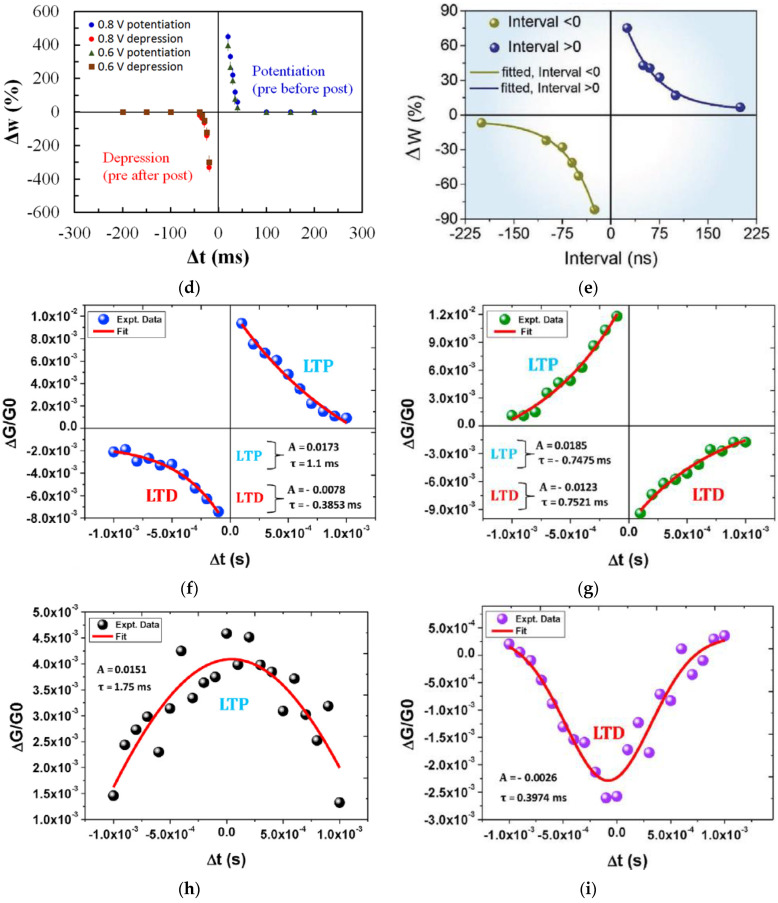
(**a**) Schematic diagram of memristor analogous to a biological synapse and STDP measurement [18]. STDP learning emulation by memristors based on various natural organic materials of (**b**) collagen [25], (**c**) bombyx mori silk [55], (**d**) honey [18], (**e**) cross-linked wool keratin [59]. (**f**–**i**) Four STDP learning rules: (**f**) asymmetric Hebbian, (**g**) asymmetric anti-Hebbian, (**h**) symmetric Hebbian, and (**i**) symmetric anti-Hebbian reported by trypsin-memristor [26].

Memory can be regarded as the strength of synaptic connections, i.e., synaptic weights. Spike-timing-dependent plasticity (STDP) is defined as the modification of synaptic weight when successive postsynaptic and presynaptic action potentials with a time interval (Δt) are applied on the neurons. The STDP is closely correlated with the relative timing of action potentials (or spikes) of the presynaptic neuron and postsynaptic neuron. The synaptic weight increases (decreases) when the presynaptic spike reaches the synapse a few milliseconds before (after) the postsynaptic spikes, which strengthens (weakens) the synaptic connection and leads to potentiation (depression) of the synapse. As shown in Figure 9a [18], in a STDP measurement, a pair of stimulation voltage pulses with different time intervals Δt between presynaptic and postsynaptic spikes is applied on the memristor to modulate the conductance of the natural organic film, with one pulse on the top electrode and the other pulse on the bottom electrode to emulate presynaptic and postsynaptic spikes, respectively. EPSC and synaptic weight are mimicked by the currents in the memristor. The change of the synaptic weight Δw is defined by Δw = (I_after_ − I_before_)/I_before_ × 100% = (G_after_ − G_before_)/G_before_ × 100% [62,63], where I_before_ and I_after_ are currents and G_before_ and G_after_ are conductance in the memristor before and after presynaptic and postsynaptic voltage pulses (V_pre_ and V_post_) are applied.

Memristors based on honey [18], trypsin [26], collagen [25], casein [34], bombyx mori silk [55], wool keratin [59], etc., have reported STDP learning behavior as shown in Figure 9b–i. When a pair of stimuli with the time internal Δt was applied on these memristor devices, similar STDP behaviors were demonstrated. When the interval Δt was greater than 0, which indicated that the presynaptic neuron stimulation occurred before the postsynaptic neuron stimulation was applied, the synaptic weight increased since the presynaptic spike induced postsynaptic depolarization and, therefore, the potentiation. In contrast, when the interval Δt was less than 0, in which the presynaptic neuron stimulation was behind the postsynaptic neuron stimulation, the synaptic weight decreased, and depression occurred. It was also observed in Figure 9b–f that the weight modification decreased when the time interval Δt between the presynaptic and postsynaptic spikes increased, and the greatest change in synaptic weight occurred when Δt was in the ±10 ms scale.

Trypsin-memristor [26] has reported four STDP rules based on the ratio of conductance $\Delta G/G_{0}$ as the modification of synaptic weight. The asymmetric Hebbian is a combination of long-term potentiation occurring at Δt > 0) and long-term depression occurring at (Δt < 0), as in Figure 9f. This rule has also been demonstrated by other natural organic materials in Figure 9b–e. Asymmetric anti-Hebbian STDP learning rule was mimicked by changing the polarity of the applied spikes as in Figure 9g. The device also mimicked the symmetric Hebbian learning rule, which generally occurs in neuromuscular junction where potentiation and depression occur at Δt ~ 0 and as Δt moves away from 0, as shown in Figure 9h. When depression occurs for all Δt values between the presynaptic and postsynaptic spikes, such learning rule is known as symmetric ani-Hebbian, which was mimicked by a trypsin-memristor, as shown in Figure 9i.

### 3.4. Spike-Rate-Dependent Plasticity

**Figure 10:**
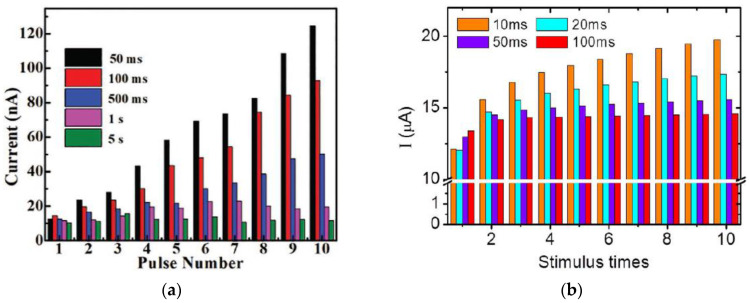

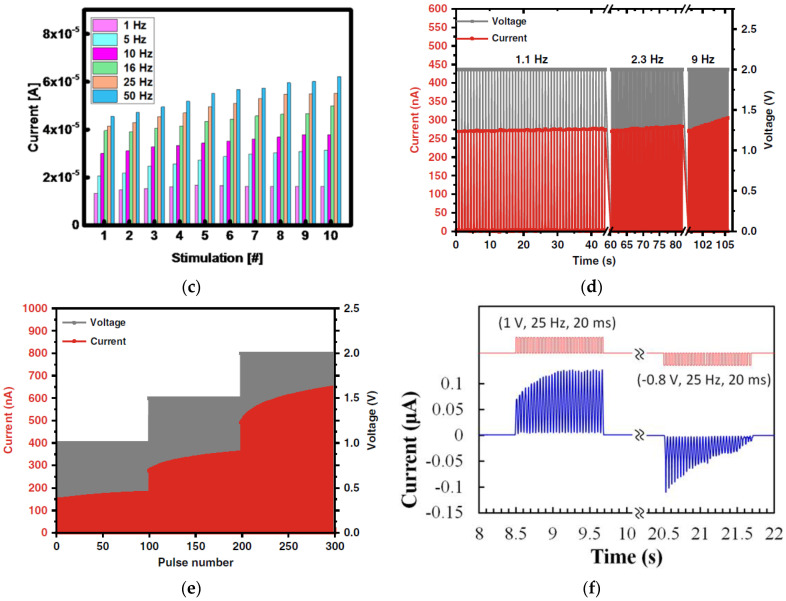
SRDP test results on various biomaterials-based synaptic devices, (**a**) cellulose-memristor [54], (**b**) chitosan-transistor [5], (**c**) collagen-memristor [25], (**d**,**e**) chitosan-memristor [3], and (**f**) honey-memristor [18].

Spike-rate-dependent plasticity (SRDP) is another important synaptic learning function in human neural networks, especially for brain cognitive behaviors [64,65]. It is the frequency-dependent plasticity, in which the synaptic weight is modulated according to the firing frequency (or rate) of the presynaptic spikes applied on the presynaptic neurons. A higher frequency or rate leads to synaptic potentiation. Unlike STDP tests in which stimulation was applied on both electrodes of a memristor that mimics presynaptic and postsynaptic neurons, in the SRDP test, stimulations, usually a voltage pulse train, is applied only on one of the electrodes to mimic presynaptic spikes with the recorded current response in the memristor to emulate EPSC and synaptic weight. For synaptic transistors, the pulse train is applied on the gate electrode. As shown in Figure 10, synaptic devices made from chitosan [5], honey [18], collagen [25], pectin [35], natural acidic polyelectrolyte [39], cellulose [54], wool keratin [59], etc., have reported the emulation of SRDP learning successfully.

A memristive device based on cellulose nanocrystals mixed with Ag nanoparticles (Ag|AgNPs-TCNC|FTO) [54] has shown SRDP when the device was tested for comparison by applying five sets of 10 voltage pulse trains with the same amplitude of −0.10 V and pulse width of 30 ms but different frequency by various intervals of 50 ms, 100 ms, 500 ms, 1s, and 5s. As shown in Figure 10a, the measured EPSC current response with each pulse number shows clearly rate-dependent, as when the interval time between the consecutive pulses was less than 500 ms, an increment in the current level was observed, while when the pulse interval was 1 s and 5 s, the recorded current response did not increase noticeably. Other natural organic memristors [3,5,18,25] demonstrated similar SRDP with rate-dependent synaptic potentiation [Figure 10b–f]. It is worth mentioning that a honey-memristor demonstrated both potentiation and depression [18], as shown in Figure 10f. When a voltage spike train with 30 repetitive stimulation pulses (amplitude: 1 V, frequency: 25 Hz, pulse width: 20 ms) was applied on the top Cu electrode, the magnitude of the currents increased, indicating a synaptic potentiation with increased synaptic weight. Following the positive voltage spike train, a negative voltage spike train of 30 repetitive pulses with the same frequency and pulse width but an amplitude of −0.8 V was applied to modulate the synaptic weight of the honey memristor. The obtained currents demonstrated synaptic depression behavior with the decreasing current magnitude.

### 3.5. Dynamic Filtering

Since the synaptic weight is activity-dependent, synapses can act as dynamic filters for information transmission [66,67]. Depending on the stimuli frequency, synapses can act as high (low) pass filters when the synapse selectively responds to high (low) frequency signals due to the short term synaptic facilitation (depression). When a biological synapse exhibits high pass filtering, a threshold must be met by the stimuli in order for an action potential to travel to the next synapse, whereas those stimuli that do not meet the threshold are blocked. Therefore, dynamic filtering function is important for neural computation as it can selectively augment the synaptic response by high frequency inputs and reduce the impact of low frequency inputs. A dynamic high pass frequency filter-like synaptic device has potential for applications in neural network and algorithm level such as non-linear autonomous learning component in neuromorphic systems [68].

**Figure 11:**
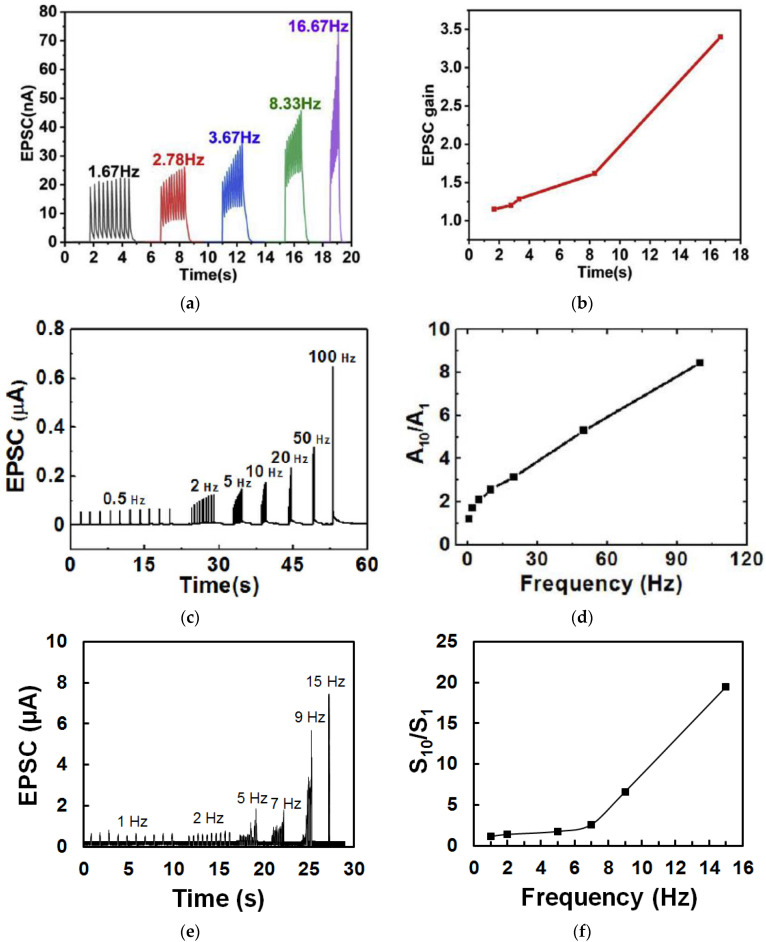
Dynamic filtering characteristics demonstrated in (**a**,**b**) gelatin [15] and (**c**,**d**) chicken albumen [43] gated synaptic transistors, and (**e**,**f**) honey-memristor [19].

Dynamic filtering has been mimicked by a memristor based on honey [19], and a synaptic transistor based on gelatin [15], sodium alginate [31], wood-derived cellulose nanopapers [32], biomass-based hydrogel [33], casein [34], chicken albumen [43], etc. During the dynamic filtering tests, a spike train with multiple voltage pulses at different frequencies is applied on one of the electrodes of the memristor or the gate of the synaptic transistor as the presynaptic stimuli, while the current flow in the device is recorded as the EPSC response. As shown in Figure 11a, from a gelatin transistor [15], in which each spike train consisted of 10 voltage pulses, it is clearly showed that the EPSC remained unchanged when the 10 presynaptic spike pulses had a low frequency of 1.67 Hz but increased rapidly with an increase of pulse frequency. Figure 11b summarizes the EPSC gain, which refers to the ratio of the amplitudes between the last EPSC peak and the first EPSC peak at different frequencies. Such increase of the EPSC gain with the increase of the presynaptic spike frequency indicates that the synaptic device acts as a high pass filter. Similar dynamic filtering has also been demonstrated by a chicken albumen [43] gated synaptic transistor and a honey-memristor [19], as shown in Figure 11c–f.

### 3.6. Spatial Summation

Synaptic integration is an important neural information process. It refers to the summation of the EPSCs in the postsynaptic neuron that were evoked by the input signals in the multiple presynaptic neurons [69]. The combined effects of excitatory and inhibitory signals from multiple inputs will determine whether an action potential will be generated in the postsynaptic neuron. Summation between neurons has two forms: spatial summation and temporal summation [70,71,72]. In spatial summation, the input spikes are applied on multiple presynaptic neurons, while the temporal summation sums the EPSC response in the postsynaptic neuron when repeated inputs are applied on the presynaptic neuron. During the test, two or more input pulses are applied on the in-plane gates of the synaptic transistor or top electrodes of the memristor, which mimics the presynaptic input terminals, with the channel conductance or memristive film conductance emulating the synaptic weight. For those two input pulses, positive input pulses emulate excitatory post-synaptic potential (EPSP) of the excitatory synapse and negative input pulses mimic inhibitory post-synaptic potential (IPSP) of the inhibitory synapse. These input stimuli could be optical pulses or electrical (voltage) pulses. In biological neurons, if the EPSC summation results in a depolarization with sufficient amplitude to raise the membrane potential above the threshold, then the postsynaptic neuron will produce an action potential. Synaptic summation has been reported by a honey-memristor [17], wood-derived cellulose nanopapers [32], a chicken albumen-gated synaptic transistor [43], a chlorophyll-a-gated synaptic transistor [60], sodium alginate transistor [31], etc.

**Figure 12:**
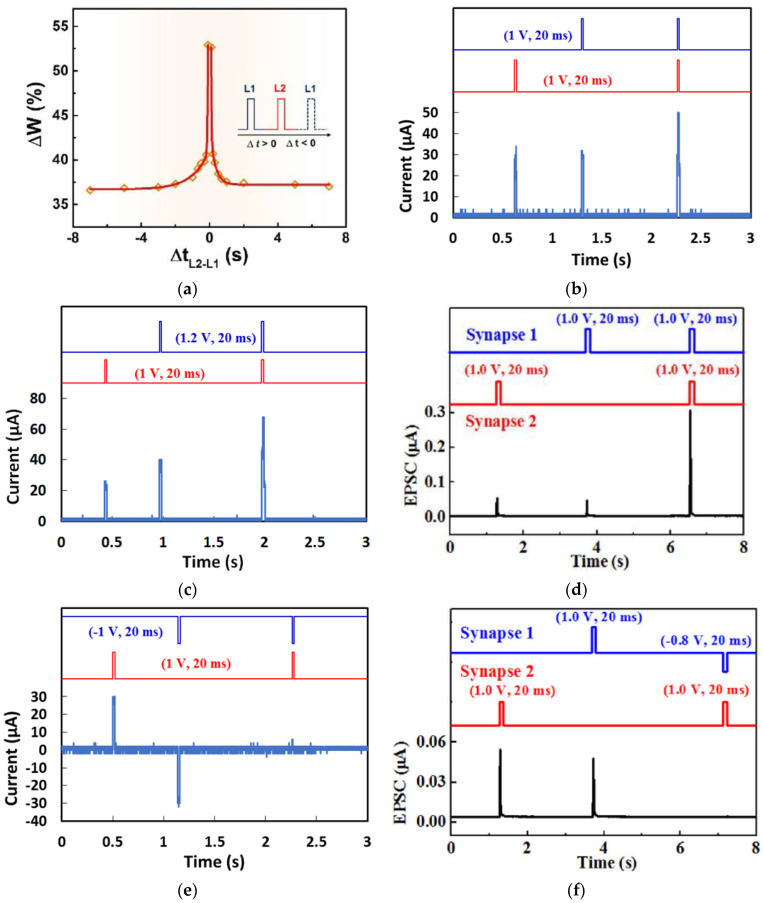
Synaptic spatial summation reported by natural organic transistors and memristors. (**a**) Chlorophyll-a-based synaptic transistor with optical input stimuli [60]. (**b**) Sublinear spatial summation and (**c**) linear spatial summation by honey-memristors [17]. (**d**) Supralinear spatial summation by chicken albumen-gated synaptic transistors [43]. (**e**,**f**) Shunting inhibition by honey-memristors [17] and chicken albumen-gated synaptic transistors [43].

Synaptic summation was demonstrated by a chlorophyll-a-gated synaptic transistor [60] when employing two presynaptic optical stimuli with an inter-spikes time interval (Δt_L2–L1_). The EPSC pulses evoked by the two optical input spikes were superimposed, which led to an increase of the final postsynaptic EPSC. The strength of spatial summation was evaluated by the synaptic weight change (ΔW), which was defined as the ratio of the peak current spike difference to the first peak current. It was found that ΔW is dependent on Δt_L2–L1_, as illustrated in Figure 12a. The maximum superimposition ΔW in the postsynaptic neuron was achieved when optical spikes 1 and 2 were applied simultaneously (Δt_L2–L1_ = 0) and reduced symmetrically as |Δt_L2–L1_| increased.

Synaptic summation in natural organic memristors and transistors by electrical stimuli were also reported. Honey-memristors [17] demonstrated both sublinear summation and linear summation when two voltage input pulses were applied. The sublinear spatial summation occurred when the amplitude (50 µA) of the final postsynaptic EPSC was smaller than the mathematical summation (60 µA) of the amplitude of the two EPSC pulses (30 µA, respectively) evoked by the two voltage input pulses with the same pulse width and amplitude, which were applied on the memristor, as shown in Figure 12b. The linear spatial summation occurred when two voltage input pulses of the same pulse width but different amplitudes were applied, in which the amplitude (68 µA) of the resulted postsynaptic EPSC was close to the mathematical summation (64 µA) of the amplitude of the two EPSCs (26 µA and 40 µA) evoked by the input voltage pulses, as shown in Figure 12c. Supralinear spatial summation was observed in chicken albumen-gated synaptic transistors [43]. When two presynaptic voltage spikes with the same pulse width and amplitudes were applied on the two gates separately, the measured postsynaptic EPSC peak (105.1 nA) was much larger than the sum (21.3 nA) of the two EPSC peaks (8.8 nA and 12.5 nA) evoked by the inputs (Figure 12d).

Inhibitory synapses also impact signal processing in the brain by limiting the flow of information and suppressing unwanted signals. When EPSP and IPSP are applied on the presynaptic neurons, shunting inhibition [73] occurs with the resulted EPSCs in the postsynaptic neuron canceled, so the postsynaptic neuron will remain silent. Such effect was also reported by honey-memristors [17] and chicken albumen-gated synaptic transistors [43], as shown in Figure 12e,f. When a positive voltage pulse (EPSP) and a negative voltage pulse (IPSP) with the same width and amplitude were applied simultaneously, shunting inhibition resulted in almost no postsynaptic EPSC in the device.

## 4. Device Fabrication

Table 1 summarizes the fabrication process of some representative synaptic devices. In general, natural organic materials are prepared by a low-cost solution-based process to form memristive devices and synaptic transistors. The natural organic materials are either in liquid form, such as cellulose hydrogel, chicken albumen, honey, etc., or in powder form dissolved in DI water, such as lignin, collagen, etc. The solution forms the natural organic film by either spin-coating or drop-casting onto the flexible or rigid substrate with pre-deposited and patterned bottom electrodes. The film needs to be completely dried in vacuum or at elevated temperate. The baking temperature and duration are specific for each organic material. Top electrodes are deposited and patterned on top of the natural organic film through a shadow mask to complete device fabrication. The materials for top and bottom electrode are also specific for each organic material. Such a solution-based microfabrication process allows devices based on natural organic materials, including honey and chicken albumen, to be fabricated on flexible substrates that are compatible with flexible microelectronics. To scale up the devices, a crossbar configuration has been applied with demonstration of natural organic synaptic device arrays based on diverse materials such as chitosan [4], zein [14], ι-carrageenan [27], wool keratin [59], and chlorophyll-a/cellulose [60], etc.

## 5. Mechanisms

The mechanisms governing synaptic current and conductance change can be classified based on transistors and memristors and natural organic materials they are made from. For electrically-driven synaptic transistors [3,4,5,15,29,30,31,32,33,34,35,36,43,56], an electric double layer (EDL) effect is responsible for current conduction, in which the migration of charged mobile carriers towards and away from the interface between the channel and natural organic film modulates the corresponding electron concentrations in the channel and, therefore, the channel conductance. The charged carriers in the natural organic films are either protons in materials such as in chitosan, albumen, maltose, etc. [3,4,5,29,30,31,32,33,34,35,36,43,56], or ions in gelatin [15], with their migration induced by drain or gate bias depending on the bias configuration. When proton or ion migration is triggered by presynaptic spikes applied on the drain or gate electrode, channel current is produced with amplitude adjusted by the concentration of protons or ions at the channel and natural organic film interface. Such a process is similar to the spike-modulated movement of the neurotransmitters in biological synapses.

For photonic synaptic transistors [39,57,60], the light-tunable synaptic plasticity is by photoexcitation in the light-sensitive natural organic film. For natural acidic polyelectrolyte [39], photogenerated electrons transfer from natural organic layer to trapped levels, while photogenerated holes are unpaired and induced by gate electric field and accumulate in the conducting channel. This hole accumulation is promoted by the large EDL capacitance induced by the high proton concentration in the light-sensitive layer, which enhances photoelectric sensitivity of synaptic transistors. After light irradiation turns off, electrons in shallow defect levels are released to recombine with holes, leading to the rapid decay of postsynaptic current. Current change in chlorophyll-a based synaptic transistor [57] is attributed to the energy transfer from chlorophyll-a to incorporated single walled carbon nanotubes. For a cellulose nanopaper-based optoelectronic synaptic transistor [60], migration of ions such as sodium ions in the film is responsible for the synaptic current.

Current conduction in memristors is mainly due to the formation of conductive paths in natural organic films. In zein [14] and lignin [24] memristors, conductive paths are carbon-rich conductive filaments formed due to the pyrolysis process induced by the local Joule heating in the film under an external bias. Such carbon-rich filaments can be ruptured by increased thermal driving due to negative bias. Conductive paths can also be formed by metal conductive filaments due to redox reaction of the metal atoms from the metal electrode such as in honey [17] and cellulose [54] memristors, or from incorporated metal nanomaterials such as carboxymethyl ι-carrageenan [28] and silk fibroin [55] with Ag nanoclusters. Metal atoms are oxidized to metal ions that migrate towards the other electrode under the electrical field and are reduced to metal atoms when reaching the other electrode. Such metal atoms accumulate between two electrodes to form metal conductive filaments for current conduction. The constituents of some natural organic materials are either charged or ionized, such as Mg ions in collagen [25] or positively charged lysine and arginine in trysin [26]. Such charged carriers drift under bias and form conductive paths in the natural organic film, analogous to neurotransmitters in biological synapses.

## 6. Future Prospects

The emulation of synaptic functions has been demonstrated by memristors and transistors with the memristive film and gate dielectric made from a variety of natural organic materials. Natural organic materials have the advantages of being renewable, abundant in nature, biodegradable, eco-friendly, etc., together with the capabilities of mimicking the synaptic plasticity and functionalities of biological synapses and neurons. Natural organic artificial synaptic devices offer a new avenue for the construction of essential hardware components for emerging neural networks and neuromorphic computing systems with energy efficiency, biodegradability, sustainable source of material, low cost fabrication, and environmentally friendly disposal, which is promising for solving the energy, sustainability, and electronic waste issues currently faced by hardware made from conventional inorganic materials. Besides these prospects, there are still opportunities for natural organic synaptic devices in more applications for neuromorphic computing. Natural organic materials have the potential to make flexible electronic devices for flexible neuromorphic systems, but so far only a limited number of materials including lignin [24], collagen [25], carboxymethyl ι-carrageenan [28], dextran [30], silk fibroin [55], wool keratin [59], etc., have been used to fabricate flexible artificial synaptic devices. Another opportunity exists in photonic synaptic devices activated by optical stimulations. Most of the current natural organic synaptic devices rely on electrical signals, which are suitable for the integration of an artificial neural network (ANN) with high density [74], but may limit the operating speed with bandwidth-connection-density trade-off [57]. Instead, photonic synaptic devices are promising for ultrafast neuromorphic computing systems [75,76,77] by advantages [60] such as interference immunity, high bandwidth, low power computation, etc. Furthermore, more investigations are needed to explore more synaptic properties demonstrated by devices based on other organic materials, such as complementary synapse by a synaptic transistor made from organic graphene-ferroelectric copolymer P(VDF-TrFE) in which the analog weight update can be positive or negative [78,79,80]. With these benefits and potential applications, more natural organic materials could be explored for photonic synaptic devices besides chlorophyll-a [57,60].

## 7. Conclusions

This paper reviews synaptic functions demonstrated by memristor- and transistor-based artificial synaptic devices made from natural organic materials, mainly carbohydrates and protein. Some elements of fundamental synaptic plasticity including short-term and long-term memory, neural facilitation, spike-timing-dependent plasticity, spike-rate-dependent plasticity, high pass and low pass dynamic filtering, and spatial summation were emulated by a variety of natural organic synaptic devices. The testing processes for each synaptic function were described, with representative testing results reported. This review provides as a useful guidance for investigating high performance synaptic memristors and transistors based on natural organic materials toward the development of sustainable, biodegradable, and eco-friendly neuromorphic computing systems in the future.

## Figures and Tables

**Table 1:** Fabrication process of synaptic devices made from some representative natural organic materials.

Ref.	Natural Organic Material	Substrate Material	Solution	Film Coating Method	Film Baking			Electrodes
					Temperature (°C)	Time (min)	Ambient	
[3]	Chitosan	Polyimide	rGO and Chitosan in DI water	Drop-casting	150	90	Ar	Ti/Au; Ti/Au
[4]	Chitosan	Glass	Chitosan and C_3_N_4_ in DI water	Drop-casting	RT	_	Air	IZO; ITO
[5]	Chitosan	Glass	rGO and Chitosan in DI water	Spin-coating	RT	_	Air	IZO; ITO
[14]	Zein	Glass	Zein in various solvents	Spin-coating	50	30	Air	Al; ITO
[15]	Gelatin	Si	Gelatin powder in DI water	Spin-coating	RT	300	Vacuum	Au; Au
[17]	Honey	Glass	Honey in DI water	Spin-coating	90	540	Air	Ag; ITO
[24]	Lignin	PET	Lignin powder in NH_4_OH and distilled water	Spin-coating	RT	48h	_	Au; ITO
[25]	Collagen	PET	Collagen powder in DI water	Spin-coating	60	90	Vacuum	Mg; ITO
[26]	Trypsin	Glass	Trypsin powder in Tris-Cl buffer	Drop-casting	RT	48h	Air	Au; FTO
[27]	ι-car	SiO_2_/Si	ι-car powder in acetic acid and distilled water	Spin-coating	RT	360	Air	Ag; Pt
[30]	Dextran	Si	Dextran in DI water	Spin-coating	70	60	Air	Au; Au

## Data Availability

No new data were created or analyzed in this study. Data sharing is not applicable to this article.
